# Carbon Materials in Voltammetry: An Overview of Versatile Platforms for Antidepressant Drug Detection

**DOI:** 10.3390/mi16040423

**Published:** 2025-03-31

**Authors:** Joanna Smajdor, Katarzyna Fendrych, Anna Górska-Ratusznik

**Affiliations:** 1 Faculty of Materials Science and Ceramics, AGH University of Krakow, al. Mickiewicza 30, 30-059 Krakow, Poland; fendrych@agh.edu.pl; 2 Lukasiewicz Research Network—Krakow Institute of Technology, 73 Zakopianska St., 30-418 Krakow, Poland

**Keywords:** carbon-based sensor, carbon nanomaterials, depression, pharmaceutical analysis, voltammetry

## Abstract

This review concentrates on the application of carbon-based materials in the development and fabrication of voltammetric sensors of antidepressant drugs used in the treatment of moderate to severe depression, anxiety disorders, personality disorders, and various phobias. Voltammetric techniques offer outstanding sensitivity and selectivity, accuracy, low detection limit, high reproducibility, instrumental simplicity, cost-effectiveness, and short time of direct determination of antidepressant drugs in pharmaceutical and clinical samples. Moreover, the combination of voltammetric approaches with the unique characteristics of carbon and its derivatives has led to the development of powerful electrochemical sensing tools for detecting antidepressant drugs, which are highly desirable in healthcare, environmental monitoring, and the pharmaceutical industry. In this review, carbon-based materials, such as glassy carbon and boron-doped diamond, and a wide spectrum of carbon nanoparticles, including graphene, graphene oxides, reduced graphene oxides, single-walled carbon nanotubes, and multi-walled carbon nanotubes were described in terms of the sensing performance of agomelatine, alprazolam, amitriptyline, aripiprazole, carbamazepine, citalopram, clomipramine, clozapine, clonazepam, desipramine, desvenlafaxine, doxepin, duloxetine, flunitrazepam, fluoxetine, fluvoxamine, imipramine, nifedipine, olanzapine, opipramol, paroxetine, quetiapine, serotonin, sertraline, sulpiride, thioridazine, trazodone, venlafaxine, and vortioxetine.

## 1. Introduction

According to the World Health Organization (WHO), depressive disorder (commonly referred to as depression) is a mental disorder characterized by a depressed mood or the loss of enjoyment or interest in activities over a long period of time. This disease affects many aspects of daily life, including work, school, and relationships with family and friends. Unlike temporary sadness or mood swings, depression is a serious illness that requires professional intervention. The WHO reports that depression affects more than 280 million people worldwide and is a leading cause of disability. It is estimated that approximately 5% of adults in developed countries suffer from depression, with women more at risk than men [1,2].

DSM-5 (Diagnostic and Statistical Manual of Mental Disorder, Fifth Edition) criteria specify that a depressive episode lasts no less than 2 weeks and patients experience at least five symptoms, including depressed mood and anhedonia (inability to feel pleasure). Other symptoms may include changes in appetite, fatigue, lack of energy, inappropriate guilt, insomnia or excessive sleepiness, difficulty concentrating, and suicidal thoughts [3]. Depending on the number and intensity of symptoms and the impact on the patient’s life, a depressive episode can be classified as mild, moderate, or severe. It can also follow different patterns, including single-episode disorder, recurrent depressive disorder (with at least two episodes), and bipolar disorder, where depressive episodes alternate with manic symptoms like euphoria, increased energy, and impulsive behavior [1,2].

These days, depression can be effectively treated, mainly with psychotherapy and/or pharmacology. Psychological treatment, such as behavioral or interpersonal therapy, is the first-line approach in the management of mild cases of depression, while patients suffering from moderate or severe depression usually require the introduction of antidepressants. However, according to a meta-analysis published by Cuijpers et al., the most effective approach in depression therapy is a combination of both psychotherapy and appropriate medications [4].

Antidepressants can be divided into several groups based on their mechanism of action. The first and most commonly prescribed group are selective serotonin reuptake inhibitors (SSRIs), such as fluoxetine, sertraline, or citalopram. Their therapeutic effect is associated with an increase in serotonin levels in the brain. There are also serotonin–norepinephrine reuptake inhibitors (SNRIs), including venlafaxine and duloxetine, which affect serotonin and norepinephrine levels. The older group of drugs is tricyclic antidepressants (TCAs), such as amitriptyline and imipramine. They are still used but cause more side effects than the newer generation of drugs. There are also monoamine oxidase inhibitors (MAOIs) (phenelzine and tranylcypromine), which require dietary restrictions because of possible interactions, and atypical antidepressants (e.g., bupropion and mirtazapine), which are used in special cases [5,6].

The quality control of antidepressants is important to ensure their safety and efficacy. The use of poor-quality drugs may result in reduced therapeutic effects, adverse reactions, or even toxicity. Accurate dosing and purity of the therapeutic substance are essential because even small variations can affect the response to treatment. Therefore, there is a need for precise, accurate, and highly sensitive analytical methods that allow the quality control of drugs available on the market. Methods to monitor the levels of drugs or their metabolites in blood or urine would also be useful in some clinical applications.

Depression is a problem that affects a large part of society. More and more people are seeking help, which translates into the increased use of antidepressants. Once administered, the drugs are either metabolized or excreted unchanged, eventually entering wastewater and potentially contaminating the environment. Antidepressant contamination can affect aquatic ecosystems by altering the behavior and reproduction of fish and other living organisms. Therefore, effective monitoring of wastewater and natural waters is needed to minimize environmental risks.

Voltammetry is a versatile electrochemical technique that can be used for both the quality control of antidepressants and environmental monitoring of their residues. This technique allows for the sensitive and selective detection of pharmaceutical compounds in pharmaceutical formulations, biological fluids, and environmental samples. The most important part of each voltammetric system is a working electrode. Various constructions can be used, and many of them are based on carbon materials, such as glassy carbon, carbon paste, graphene, boron-doped diamond, etc. Carbon-based materials offer several advantages, including high electrical conductivity, wide potential windows, good chemical stability, and the ability to be modified for improved sensitivity and selectivity. These properties make carbon electrodes particularly suitable for electrochemical applications, including the detection of antidepressants in pharmaceutical and environmental samples.

## 2. Principle of Electrochemical Sensor

**Figure 1:**
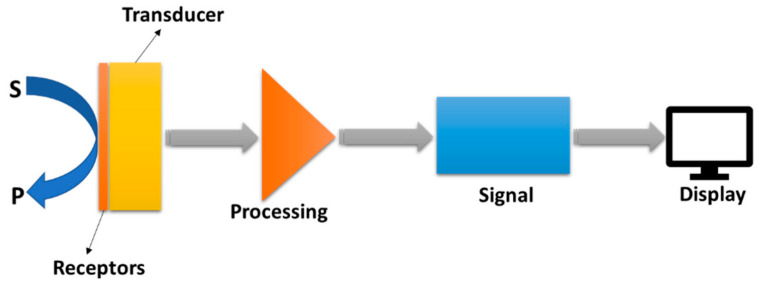
A schematic diagram illustrating the major components of a standard sensor. Reprinted from *Electrochemical Sensors and Their Applications: A Review*, Chemosensors 2022 [9].

According to the IUPAC (International Union of Pure and Applied Chemistry), a chemical sensor is defined as “a device that converts chemical data, ranging from the concentration of a single sample component to complete composition analysis, into an analytically usable signal” [7]. This device typically comprises two main functional components: a receptor and a physicochemical transducer (Figure 1). The receptor, through highly specific interactions with the analyte, converts chemical information about a sample into a form of energy, while the transducer then transforms this energy into the form of an analytical signal with a measurable value [8]. Chemical sensors, based on the operating principle of the transducer, can be classified as (1) optical, converting changes in optical phenomena (i.e., absorbance, reflectance, luminescence, and fluorescence) resulting from the receptor–analyte interaction; (2) electrochemical, converting electrochemical interaction between the analyte and the electrode; (3) mass, transforming the changes in mass caused by the accumulation of the analyte on the receptor surface; (4) thermal, based on the heat effects associated with a particular chemical reaction or adsorption involving the analyte; (5) and magnetic, based on the alteration of the paramagnetic properties of the analyte [7].

Among a wide group of electrochemical sensors [9,10], voltammetric sensors constitute a significant analytical tool because of their high selectivity, excellent sensitivity, wide detection range, low cost, and ease of miniaturization and realization of automatic measurements. The voltammetric sensors provide information about an analyte by measuring the current as a function of the varying potential that causes oxidation or reduction in electroactive species at the surface of the working electrode. Consequently, the resultant current is proportional to the concentration of the electrochemical species [11].

As electrochemical reactions take place on the surface of the working electrode, its surface characteristics play a crucial role in the efficiency of the voltammetric sensor. Working electrodes are typically made from chemically stable materials with a high electrical conductivity to efficiently transfer electrons during an electrochemical reaction. Common working electrodes can be made from a variety of materials, ranging from noble metals like gold or platinum to inert carbon materials such as glassy carbon, boron-doped diamond, pyrolytic carbon, various conductive polymers, and mercury drop and film electrodes. 

Currently, to improve the performance of voltammetric sensors, the working electrode is typically modified with a chemically stable material capable of enhancing the electron transfer rate, exhibiting electrocatalytic properties, reducing the detection overpotential of the analyte, and enabling its selective binding (preconcentration) at the electrode surface [12]. By chemically modifying the electrode surface, the performance of the sensor can be tailored to meet the specific needs of a given application, offering improved precision, reliability, and sensitivity [13]. For this purpose, advanced nanostructures with unique physical and chemical characteristics, like metal-based and carbon-based nanomaterials, metal–organic frameworks, conducting polymers, molecularly imprinted polymers, zeolites, etc., are still being investigated and utilized [14,15,16]. As a result, a wide spectrum of CMEs (chemically modified electrodes) have been fabricated and implemented in the trace analysis of heavy metals and organic pollutants, the detection of biomolecules, environmental monitoring, food quality control, and pharmaceutical analysis. 

## 3. Carbon-Based Electrochemical Sensors of Antidepressant Drugs

Carbon and its derivatives are considered exceptional materials in electrochemistry because of their remarkable properties, such as low cost, relatively wide potential range, chemical inertness, rich surface chemistry, and suitability for different types of analytes. They play a crucial role both as working electrode material and as a modifying agent of working electrode that enhances its performance [17]. In the voltametric analysis of antidepressant drugs, carbon-based electrodes, such as glassy carbon electrodes (GCEs), carbon paste electrodes (CPEs), screen-printed carbon electrodes (SPCEs), and boron-doped diamond electrodes (BDDEs) are commonly used. 

### 3.1. Glassy Carbon Electrode

**Figure 2:**
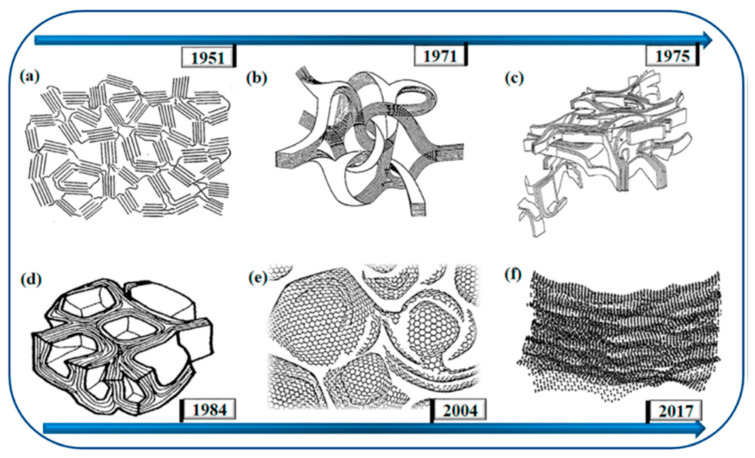
Schemes presenting structural models of glassy carbon proposed over years by (**a**) Franklin; (**b**) Jenkins and Kawamura; (**c**) Ban; (**d**) Shiraishi; (**e**) Harris; (**f**) Jurkiewicz [18]. Reprinted from ref. [18], with permission from Elsevier.

Glassy carbon (GC), also known as vitreous carbon, is a material fabricated through the carbonization of thermosetting resins (such as phenolic resins, polyfurfuryl alcohol, or polyarylacetylene) under an inert atmosphere. The resulting material is composed of low-ordered graphite layers with sp^2^ carbon organized into a hexagonal pattern. Carbon atoms within the layer are covalently bonded, while the layers interact with each other through weak van der Waals interactions. A characteristic of GC is a turbostratic structure, which means that the graphitic layers are randomly positioned and rotated toward each other, resulting in low structural order [18]. Over the years, scientists have attempted to develop a detailed model of the structure of GC that would be consistent with the physicochemical properties of the material. According to the literature, the most up-to-date models that give the best explanation of the GC structure are those of Harris or Jurkiewicz [18]. Harris’s model presumes that random fragments of curved carbon layers are arranged into an imperfect fullerene-like structure, leading to the formation of closed pores (Figure 2e). This would explain GC properties such as low density and poor permeability and reactivity. This theory claims that there are also non-hexagonal rings within the structure, which explains the occurrence of carbon layer curvature [19]. Jurkiewicz’s research, conducted using wide-angle neutron scattering and molecular dynamics simulations, confirmed the fullerene-like structure of GC, which is consistent with Harris’s model [20].

As mentioned, GC is characterized by good thermal stability, chemical inertness, a large pore volume, and impermeability to gases and liquids. Other characteristic properties of this material are high stiffness and hardness, high surface area, good electrical conductivity, and biocompatibility. Thus, GC is used in applications like electrochemical sensors, electrochemical devices for wastewater treatment, energy storage, heart valves, scaffolds for tissue regeneration, or neural implants [18,21].

Of particular interest from the perspective of this review is the use of GC as an electrochemical sensor. Glassy carbon electrodes (GCEs) are widely used for the detection of various types of chemical compounds and biomolecules for the pharmaceutical industry, food and clinical analysis, and the monitoring of environmental pollution. Studies have reported the use of bare GCEs for electrochemical analysis, but there are also many studies on the surface modification possibilities of GCEs. Surface modifiers such as carbon nanomaterials, metal and metal oxide nanoparticles, and organic compounds can improve parameters such as selectivity, accuracy, or sensitivity. To improve the electrochemical performance of GCEs, techniques such as mechanical polishing; chemical, electrochemical, and plasma treatments; or ultrasonication can be used. They are applied for surface cleaning and activation, resulting in the incorporation of functional groups into the GCE surface. This improves both the electroactive surface area and the electrochemical performance of the sensor [18,22].

A literature search revealed that GCEs were utilized for the voltammetric determination of some antipsychotic drugs, such as opipramol, aripiprazole, citalopram, and quetiapine (Table 1). Opipramol was detected using two techniques: DPV (Differential Pulse Voltammetry) and OSV (Osteryoung Square-wave Voltammetry) in samples of serum and urine. An irreversible, diffusion-controlled oxidation reaction was the basis of the analytical signal. Based on the obtained results, a mechanism involving the oxidation of the azepine and piperazine rings of opipramol was proposed by the authors. The LOD for the developed method was equal to 2.70 × 10^−7^ M and 3.10 × 10^−7^ M for DPV and OSV, respectively [23]. Merli [24] proposed the AdSV (Anodic Adsorptive Stripping Voltammetry) method for the determination of aripiprazole on GCEs in tablets and urine within a concentration range of 4–40 µg L^−1^ and achieved an LOD (limit of detection) equal to 1 µg L^−1^. The GCE area was determined to be 0.0203 cm^2^, which is close to the geometrical value. The intra- and inter-day precisions estimated for this electrode were equal to 0.24% and 1.52%, respectively. A similar method was described by Asangil [25]. The researcher used DPAAdSV (Differential Pulse Anodic Adsorptive Stripping Voltammetry) (LOD = 0.14 µM) and SWAAdSV (Square-wave Anodic Adsorptive Stripping Voltammetry) (LOD = 0.11 µM) for aripiprazole quantification in serum and urine. Recoveries calculated based on samples’ measurements were within the range of 95.0–104.6%. The intra- and inter-day precisions for the presented method were estimated to be 5.85–8.94% and 9.07–11.17%. Fluctuations of the peak potential were smaller and were equal to 2.32–5.67% (intra-day) and 4.76–7.98% (inter-day). A paper reporting the irreversible, adsorption-controlled electro-oxidation of citalopram on GCEs was published by Madej et al. [26]. The developed method has been successfully applied for the determination of citalopram in pharmaceuticals, tap water, river water, and wastewater, with a detection limit as low as 0.036 µM. The influence of potential interfering agents has been investigated by authors. The results proved the high selectivity of the method during the determination of samples such as pharmaceuticals and water. In the case of biological samples (CRMs of serum and urine), the impact of the matrix on the voltammetric signal was visible. Quetiapine, another antipsychotic belonging to the group of dibenzodiazepine derivatives, was quantified by DPV and OSV methods using a GCE as the working electrode [27]. The linear response was obtained over the concentration range 4 × 10^−6^–2 × 10^−4^ mol L^−1^, and the method was validated by an analysis of samples such as tablets, serum, and urine. Samples of pharmaceuticals and urine were measured without any preparation, while serum was treated with acetonitrile in order to remove proteins that might interfere during the measurement. The authors calculated the RSD% (Relative Standard Deviation) to express the repeatability of the peak current for different types of samples, supporting electrolyte, serum, and urine. In the case of DPV measurements, the results revealed that the highest inter-day precision was achieved for the electrolyte (RSD = 0.225%), and the lowest was achieved for urine (RSD = 1.32%). For OSWV, the lowest precision was achieved during QTP analysis in urine (RSD = 0.97%). Recoveries were also calculated and were equal to 99 ± 3% for DPV and 100 ± 1% for OSWV. The LODs for both the proposed techniques were equal to 4.01 × 10^−8^ mol L^−1^ for DPV and 1.33 × 10^−7^ mol L^−1^ for OSV.

### 3.2. Boron-Doped Diamond Electrode

Diamond is a semiconductor, so despite its many advantages (e.g., chemical inertness, exceptional hardness, low friction, and high carrier mobility), it cannot be used as an electrode material. However, conductivity in diamond can be achieved by incorporating charge carriers into the structure, such as boron atoms. The resulting material is called boron-doped diamond (BDD) and, in the literature, is described as a semi-metallic or “metal-like” structure with excellent electrochemical properties [28]. Therefore, BDD films are readily used in the construction of electrochemical sensors. The main advantages of BDD-based electrodes are as follows: (a) wide potential window (in aqueous media ~3–3.5 V, in non-aqueous media ~5–7.5 V); (b) low background current associated with the low capacitance of BDD; (c) wide spectral transparency window including the UV-Vis and far-infrared regions (important for spectroelectrochemical measurements); (d) ability to modify the electrochemical properties of the electrode surface by varying the boron concentration, surface termination, and orientation and by incorporating sp^2^ carbon impurities; (e) biocompatibility and resistance to biofouling, making them suitable for medical applications [28,29,30]. 

As mentioned above, the electrochemical properties of BDD can be influenced by a few parameters. The first is the boron doping level, which affects the conductivity of the material. As the boron doping is increased, BDD changes its nature from an insulator to a metal. Increasing the B/C ratio in BDD leads to a decrease in the potential window and the peak separation of the redox pair [Fe(CN)_6_]^3−/4−^. On the other hand, a higher B/C ratio enhances charge transfer during the initial stage of oxidation of certain organic compounds, resulting in a lower LOD. The literature reports that the optimum boron concentration for electrochemical applications is >10^20^ atoms cm^−3^. A technique that allows the fabrication of BDD with desirable B concentration is microwave chemical vapor deposition (MW-CVD) [28].

The second parameter that influences the electrochemical performance of BDD is the sp^3^/sp^2^ carbon ratio in the structure. Pure diamond consists of non-active, catalytically inert sp^3^ carbon. Diamond doping with boron is usually associated with the incorporation of sp^2^-bonded carbon impurities, affecting the electroanalytical performance of the BDD electrode. Compared to sp^3^, sp^2^ carbon has a higher density of electronic states, allowing faster electron transfer. In addition, sp^2^ carbon acts as a catalyst to support redox reactions by providing adsorption sites for reactants and reaction intermediates. On the other hand, sp^2^ carbon reacts with water and oxygen to form oxygen-containing functional groups, resulting in a reduction in the electrochemical potential window and a higher background current, which are undesirable. Currently, techniques such as laser ablation could be used to incorporate sp^2^ carbon into specific regions of the BDD in a controlled manner to retain favorable properties without significant degradation of the potential window and background current. In general, in electrochemical applications, the level of sp^2^ carbon surface impurities should be controlled to achieve optimal properties of the BDD electrode [28]. 

The last important parameter affecting the electrochemical properties of BDD is the surface termination. The typical types of diamond surface termination are oxygen and hydrogen. H- and O-terminated BDDE exhibit different properties. For example, some analytes can only be detected on a certain type of BDDE (oxalic acid can be detected on H-terminated BDD, while no signal is observed on O-terminated BDD), and others can achieve better signal separation, allowing their simultaneous determination (dopamine and ascorbic acid can be easily oxidized on O-terminated BDD, while their signals overlap on H-terminated BDD). CVD-manufactured BDDs are those with H-termination, but the surface termination can be easily modified by various chemical reactions and processes [29]. 

BDDE has been used to determine antidepressants such as paroxetine, duloxetine, and fluoxetine via electro-oxidation (Table 2). In the case of duloxetine and fluoxetine, BDDE was subjected to cathodic and anodic pretreatment prior to the experiments in order to obtain two types of surface termination (O and H). Then, it was investigated which termination was more suitable for certain analytes. When it comes to duloxetine, both types of BDDE allowed to obtain irreversible oxidation peak around 1.2 V, but the cathodically pretreated BDDE provided more reproducible results and a higher and better-defined peak. The authors assessed the selectivity of the method based on the evaluation of the impact of potential interfering agents (such as organic compounds and inorganic cations and ions) on the duloxetine signal. According to the results, anions and cations did not affect the signal in the tested range of concentration. In the case of organic compounds, their effect was also negligible and did not interfere with the measurement. Duloxetine determination using DPV (LOD = 5.87 nmol L^−1^) and SWV (42 nmol L^−1^) techniques in samples of lake, river, and tap water was performed [31]. The authors performed analyte determination using DPV (LOD = 5.87 nmol L^−1^) and SWV (42 nmol L^−1^) techniques in samples of lake, river, and tap water. For fluoxetine, however, better measurement conditions were obtained with O-terminated BDDE, using the SWV technique [32]. Fluoxetine quantification was possible within a concentration range of 3.2–162 µmol L^−1^, and the LOD value was equal to 0.3 µmol L^−1^. The applicability of the method was proven by the measurement of different weight loss products in the form of capsules (containing substances such as bupropion, furosemide, spirulina, sinetrol, chitosan, caffeine, Camellia sinensis, and Garcinia cambogia). Sample solutions for analysis were prepared simply by weighing 1.0 g of powder and diluting it in 10 mL of the supporting electrolyte. Recoveries calculated based on samples’ measurements were within the range of 98–112% for four types of samples, which indicates the high accuracy of the method. However, in the case of products containing chitosan, a strong matrix effect was observed, and the recovery value was equal to 65 ± 3%. The last described analyte determined using BDDE was paroxetine, belonging to the group of SSRI antidepressants. Paroxetine was electro-oxidized using the SWAdSV method within the concentration range of 7.0 × 10^−7^–3.5 × 10^−6^ M. The authors suggest that the anodic response of paroxetine might be a result of alkoxybenzene moiety oxidation. The developed method allowed for rapid and sensitive quantification of the drug in tablets with a recovery of 99.58%. The repeatability and reproducibility of the peak current, expressed as %RSD, were equal to 0.72 and 1.88%, respectively [33].

## 4. Carbon Nanoparticle (CNP)-Modified Electrodes

The various forms of carbon nanoparticles (CNPs) have garnered significant attention from researchers because of their unique electronic and physiochemical properties, especially their electrochemical activity. As sensing material, CNPs can be characterized by their large specific surface area, high electrical conductivity, large adsorption ability toward analytes, electrocatalytic activity, antifouling resistance, chemical stability, nontoxicity, and biocompatibility [34,35]. The capability of CNPs to create a strong covalent bond with various materials makes them suitable for the development of efficient hybrid nanocomposites for electrochemical sensing applications. A wide spectrum of CNPs, including graphene, graphene oxides (GOs), reduced graphene oxides (rGOs), single-walled carbon nanotubes (SWCNTs), and multi-walled carbon nanotubes (MWCNTs), have been applied as modifier materials in electrochemical sensing of antidepressant drugs to facilitate electrochemical reactions due to the increased surface area, improved selectivity, stability, and enhanced surface kinetics [36].

### 4.1. Graphene and Graphene Oxide

**Figure 3:**
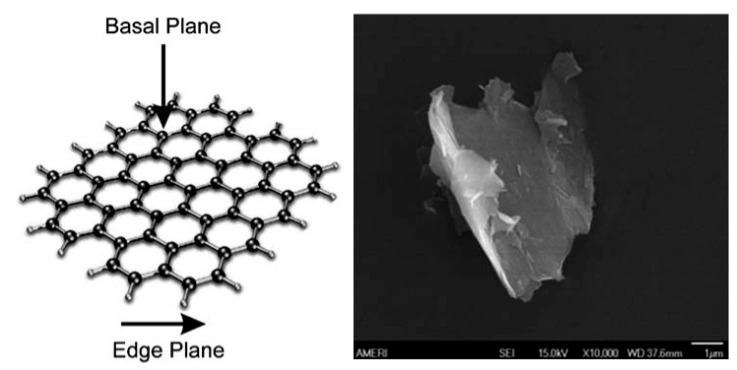
A conceptual schematic model of the structure of graphene, indicating its basal and edge plane-like sites, and an SEM image of a single atomic layer of graphite, known as graphene. Note that in reality, the graphene utilized in the majority of work is 1–4+ layers thick. Reprinted from ref. [48], with permission from Royal Society of Chemistry.

Graphene, a two-dimensional nanomaterial made up of a single layer of carbon atoms with a unique honeycomb lattice structure, has gained significant attention due to its remarkable electronic and electrocatalytic properties (Figure 3) [37,38]. Not only is it the thinnest and strongest material ever made, but it is also characterized by high thermal conductivity (5000 W m^−1^ K^−1^) [39] and transparency (97.7%) [40], superior carrier mobility at room temperature (200,000 cm^2^ V^−1^ s^−1^) [41], good electrical conductivity (2000 S m^−1^) [42], extremely high theoretical specific surface area (2630 m^2^ g^−1^) [43], excellent environmental compatibility, high mechanical strength (Young’s modulus of 1 TPa) [44], low production cost [45], and high adsorption capacity for organic and inorganic molecules [46,47]. 

**Figure 4:**
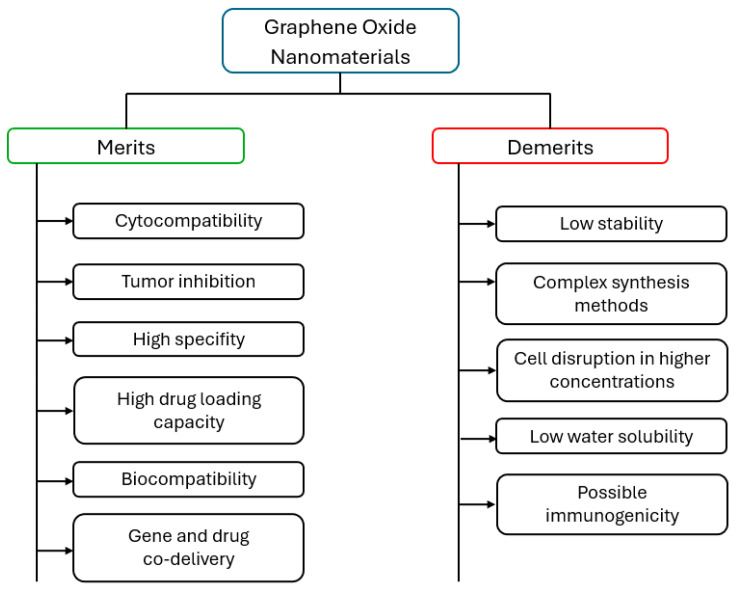
Merits and demerits of graphene oxide nanomaterials.

Although there are various methods for synthesizing graphene, the chemical oxidation of graphite to graphene oxide (GO) followed by its reduction to graphene remains one of the most effective techniques to produce large quantities of graphene for electrocatalytic applications. GO reduction can be achieved through chemical, thermal, or electrochemical methods [49,50,51]. Among these, the electrochemical approach is preferred for its simplicity, speed, environmental friendliness, and efficiency, as it does not require high temperatures like the thermal method or toxic reductants, such as hydrazine, which are used in chemical reduction. The electronic properties of graphene can be modified by combining it with transition metal oxides like ZnO, NiO, Co_3_O_4_, MnO_2_, TiO_2_, and RuO_2_. These graphene–metal oxide composites are of great interest in energy storage, electrochemical sensors, and biosensor applications due to the synergy between the conductive graphene and the electrocatalytic metal oxide particles [52,53]. Among various graphene-based nanomaterials, reduced graphene oxide (RGO) is particularly popular in electrochemical applications due to its excellent redox activity, specific capacitance, and reversibility [54]. Several synthesis methods, including co-precipitation, hydrothermal synthesis, thermal decomposition, and chemical reduction, have been used to create RGO composites with metal oxides [55,56]. However, challenges persist with these methods, such as difficulty in controlling particle size due to nanoparticle agglomeration on the GO sheets. Some of the merits and demerits of graphene oxide nanomaterials used in the field of electroanalytical application for highly sensitive pharmaceutical analysis are presented in Figure 4.

Due to the exceptional properties of graphene and its derivatives, these materials are widely used in the development of electrochemical sensors and biosensors. The electrochemical sensing process typically involves direct electron transfer between the graphene-based sensing platform and an electroactive species, which could be the target molecule itself or a related molecule whose electrochemical signal can be correlated to the target. Graphene and its derivatives are favored for such applications because of their high surface area and excellent electron conductivity, which enhance electron transfer efficiency and result in stronger signal outputs. In some instances, target molecules can be detected at lower applied potentials. Furthermore, surface modifications on graphene and its derivatives can increase their affinity for target molecules, minimizing interference effects. As a result, graphene-based sensors offer improved sensitivity and better performance in complex real samples compared to bare GC electrodes.

Recent studies have shown that electrodes modified with different graphene forms and their combinations can be used in the voltammetric analysis of psychiatric drugs (Table 3).

**Figure 5:**
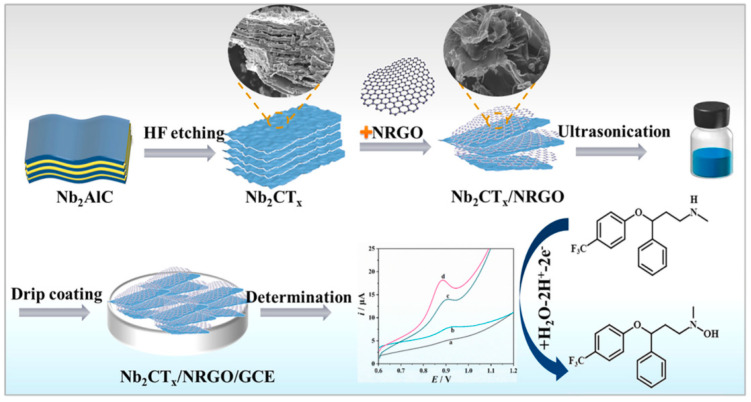
Preparation of Nb_2_CT_x_/NRGO/GCE and detection of FLX. Lines a–d: FLX oxidation peaks on: bare GCE (a), Nb2CTx/GCE (b), NRGO/GCE (c) and Nb2CTx/NRGO/ GCE (d). Reprinted from submicromolar electrochemical sensing platform for fast fluoxetine quantification based on Nb2CTx MXene and nitrogen-doped graphene oxide nanocomposites. *J. Alloys Compd*. 970, pages 172557, A. Chen, Y. Wei, D. Tuo, C. Zhou, S. Shi, N. Tang, Q. He, J. Liu, 2024, with permission from Elsevier [64].

One of the most popular graphene materials used as a component of electrode modification layer is reduced graphene oxide, mostly due to the simplicity of the preparation of a modifier solution. For example, nanocomposites of RGO and metal nanoparticles or metal oxides were currently used in voltametric analysis. A combination of RGO and Co_3_O_4_ was applied towards highly sensitive serotonin determination in pharmaceuticals and blood serum samples. The usage of this kind of modification results in achieving a detection limit of 48.7 nM and a high sensitivity of 2.2 mA mM^−1^ cm^−2^ [57]. Demonstrated sensor exhibits very good recovery values for the determination of serotonin directly from human blood serum samples, without any sample preparation pretreatments. In the next paper, a combination of RGO with nickel–cobalt oxide microspheres was prepared and placed on the pencil graphite electrode for venlafaxine determination in the pharmaceuticals and human plasma. The obtained detection limit was measured as 3.4 nmol L^−1^, and recovery values for venlafaxine determination in the real samples ranged from 96.0 to 100.7%. The use of this type of modification allowed the elimination of the influence of interference on the signal from the analyte [58]. Another type of proposed RGO usage was to mix it with palladium nanoparticles to develop a sensor sensitive to desipramine. Its determination was based on the process of electrochemical oxidation on the surface of the glassy carbon electrode modified with an RGO/PdNP suspension. The authors proved that the usage of this prepared electrode allows the determination of the antidepressant with a limit detection of 1.04 nmol L^−1^, and the sensor was successfully applied for determination in urine samples [59]. The process for quantifying the antidepressant is based on the reversible electrode oxidation of the electrochemically generated desipramine dimer. This feature is particularly noteworthy due to the higher anodic peak current observed in this electrochemical process compared to other peak currents in the desipramine voltammetric profile, potentially enhancing the sensitivity of the analytical method. For another type of RGO nanocomposite (with gold nanoparticles), laser-assisted ablation was necessary to create a new type of modification suspension. This fabricated LI-AuNP-rGO/GC electrode was used for alprazolam determination with a wide linearity range and a detection limit of 0.3 µg L^−1^. The authors used the proposed sensor for alprazolam determination in spiked beverage samples with good reproducibility and repeatability of the measurements [60]. The analytical performance demonstrated the potential of the proposed sensor as an alternative method, or even a screening tool, for detecting suspected alprazolam spiking at crime scenes. Not only metal oxides or nanoparticles can be incorporated in the composite layers consisting of RGO for voltametric measurements. Molecules such as phosphotungstic acid can be a part of modification layers, for example, in highly sensitive paroxetine determination. It was proven that this mixture placed on the pencil graphite electrode facilitates the determination of paroxetine, enabling a detection limit of 9.0 × 10^−10^ M. The authors used this electrode for real sample measurements (tablets, human serum, and urine), achieving recovery parameters in the range of 97.6–101.6% [61]. Reduced graphene oxide can also be a component of carbon paste electrodes mixed with β-Ni(OH)_2_ and ionic liquid. The combination of these modifiers leads to excellent electrocatalytic activity and voltammetric performance for sulpiride sensing. The sensitivity for determination was over twice as high as that of the unmodified CPE. The sensor demonstrated by Mohamed et al. was characterized by the authors with high reproducibility, selectivity, and sensitivity, along with advantages such as time efficiency, simplicity, and cost-effectiveness. Furthermore, the proposed electrochemical sensor was successfully applied for the quantitative detection of the analyte in tablets, urine, and plasma [62]. In another work, the AuNPs@GRP nanocomposite was synthesized in a single step using GO and gold salt and placed on the surface of the glassy carbon electrode. This prepared sensor was successfully used for vortioxetine determination in concentrations as low as 50.0 nM. The key advantages of the AuNPs@GRP/GCE, according to the authors, include high sensitivity, selectivity, reproducibility, a broad linear range, and a low detection limit. Considering these parameters, the proposed sensor demonstrates exceptional analytical performance for the determination of vortioxetine [63]. In the next study, a composite electrochemical sensor was developed for the detection of fluoxetine by etching and synthesizing niobium carbide (Nb_2_CTx) material, which was then ultrasonically combined with nitrogen-doped reduced graphene oxide (NRGO) (Figure 5). The synergistic Nb_2_CTx/NRGO/GCE exhibited superior electrochemical performance, including enhanced electroactive area, improved electron conduction, and better electrocatalytic ability compared to the bare GC electrode, making it suitable for fluoxetine (FLX) detection with a detection limit of 0.34 µM. This sensor offers a wider linear range and a lower detection limit compared to other electrochemical sensors for FLX detection, making it highly practical. Furthermore, the sensor demonstrated good reproducibility, repeatability, and stability [64]. 

Another electrode proposed for the determination of sulpiride drugs was a pyrolytic graphite electrode modified with graphene oxide and β-cyclodextrin. Such a nanosensor was capable of sulpiride determination, with levels as low as 2.83 × 10^−9^ M and an accumulation time of 30 s, and was successfully applied for its determination in drug analysis and biological samples [65]. The proposed method was characterized by excellent recovery values during sulpride determination in urine samples, without any interference effects from the sample matrix. Other forms of graphene, such as graphene nanoplatelets dispersed in Nafion, were successfully used for highly sensitive quetiapine determination, reaching a limit of detection equal to 22 nM and a broad linear range from 100 nM to 10 µM. The authors proved its usability by performing analysis in the urine samples and pharmaceutical formulations, with good recovery parameters [66]. Also, graphene nanoparticles were incorporated in voltametric assays. The modification layer consisted of graphene nanoparticles and molecular imprinting polymers (MIPs) placed on the surface of the platinum electrode and was used in sertraline determination assays. The usage of MIP modification connected with PtNPs significantly improves the response of the electrode for sertraline. The obtained detection limit was equal to 7.0 × 10^−9^ mol L^−1^, and the proposed sensor was capable of selective sertraline detection in human serum samples, with excellent recovery values in the range of 98.2–103.5% [67]. Another novel sensor for the voltammetric detection of citalopram (CTL) in solution has been developed, featuring a gold–palladium (Au-Pd) bimetallic nanoparticle-decorated graphene (GR)-modified gold electrode. This combination of GR and Au-Pd nanoparticles on the gold electrode surface demonstrates strong electrocatalytic activity for CTL oxidation, offering a low detection limit with a value of 0.049 µM, high selectivity, and good reproducibility. The electrochemical sensor was effective for the determination of citalopram in tablets and human plasma samples, providing satisfactory results [68]. In another study, a simple and sensitive electrochemical sensor for the detection of carbamazepine (CBZ) in clinical samples based on a GO/g-C_3_N_4_ composite film was introduced. The modified electrode significantly enhanced the anodic current response for the oxidation of carbamazepine compared to GO and g-C_3_N_4_ alone. The proposed sensing platform demonstrates a low detection level (10.5 nM) with high sensitivity and good selectivity. Additionally, the method offers a straightforward sensor fabrication process for determining CBZ in human urine and pharmaceutical samples, with an acceptable recovery rate [69]. Graphene and its derivatives are also used in voltammetry as the components of paste electrodes. For example, duloxetine hydrochloride (DXT) was successfully measured on a graphite paste electrode using linear sweep and square-wave voltammetry [78]. The process of DXT oxidation on a working electrode surface was found to be irreversible and controlled by diffusion. The probable scheme of the reaction mechanism was proposed, and the DXT limit of detection was measured and calculated as 3 × 10^−9^ M. Another carbon paste electrode, proposed in this case for the highly sensitive determination of escitalopram (ESC), was made of graphite powder mixed with chloranil and nickel nanoparticles [79]. Thanks to the use of this electrode, ESC was successfully measured in tablets and human urine with very good recovery parameter values. ESC determination in controlled conditions results in obtaining limit of detection values as low as 2 × 10^−7^ mol L^−1^. Also, metal oxides, such as aluminum oxide, can be used as modifiers of paste electrodes [81]. In this case, the oxidation of the aripiprazole (ARP) was observed using Square-wave Anodic Adsorptive Stripping Voltammetry. This method offers a fast, highly sensitive, selective, low-cost, and straightforward approach for analyzing ARP in tablets and spiked human serum samples. Additional benefits include a wide linear range (0.03−8.0 µM), a low detection limit (0.006 μM), excellent repeatability (RSD = 1.8%), good reproducibility (RSD = 2.9%), and good stability. Furthermore, no complex sample preparation is needed for serum samples. 

### 4.2. Carbon Nanotubes

**Figure 6:**
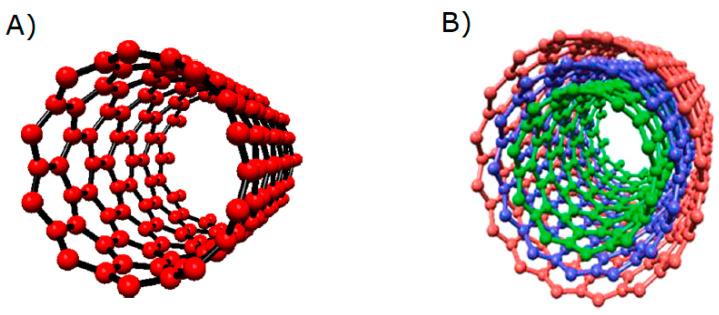
Surface and internal view of (**A**) single-walled carbon nanotube and (**B**) multi-walled carbon nanotubes. Reprinted from ref. [86], with permission from Elsevier.

Carbon nanotubes (CNTs) are unique tubular nanostructures consisting of a flat graphene sheet of carbon atoms bonded to sp^2^, arranged in a honeycomb lattice, and rolled into a cylinder with a nanometric diameter and length up to several centimeters [82]. Carbon nanotubes are usually classified into one of two groups: single-walled carbon nanotubes (SWCNTs) and multi-walled carbon nanotubes (MWCNTs) (Figure 6). A SWCNTS can be visualized as a seamless hollow cylinder (diameter from a fraction of a nanometer to several nanometers [82]) of graphene sheets, arising from a rolling a single atomic layer of crystalline graphite, while MWCNTs constitute a concentric and closed arrangement of up to several tens of SWNCTs with a diameter typically around 10–20 nm and an interlayer separated by a distance of approximately 0.34 nm [83]. Depending on the intended application, CNTs can be produced using many different synthesis methods, including carbon arc discharge, chemical vapor deposition (CVD), plasma-enhanced chemical vapor deposition (PECVD), and laser ablation [84]. Current knowledge enables the possibility of functionalizing, filling, doping, chemically modifying, separating, and manipulating individual CNTs, resulting in their wide application in nanotechnology engineering [85].

CNTs are of great interest to scientists and engineers because of their unusual properties, which arise from the planar sp^2^ bonding of carbon atoms that is characteristic of graphite. The expectational features of CNTs involve a high surface area, a high aspect ratio, and impressive material properties, such as high mechanical strength, good electrical and thermal conductivity, optical absorption, emission, and photoluminescence properties [87]. The broad spectrum of unconventional characteristics of CNTs led to their application in, e.g., lithium-ion battery technologies, hydrogen storage, fuel cells, supercapacitors, drug delivery systems, membranes for wastewater remediation, composite materials (additives to hydrogels, epoxy resins, ceramics, and polymers), and chemical sensors [85,86]. Their preparation and functionalization methods and their development are still of interest to many scientists [88,89,90,91]. 

In CNT-based sensor technology, next to gas sensors and biosensors, electrochemical sensors occupy a special space, particularly voltametric ones. The unquestionable success of the implementation of CNTs for electroanalytical applications is closely related to their excellent conductivity, electrocatalytic activity, good electron transfer, high chemical stability, very large surface area to volume ratios, and large aspect ratios. As a result, CNT-modified electrochemical sensors can be characterized by higher sensitivity, a lower limit of detection, faster transfer kinetic, and a wider potential window compared to the traditional carbon electrodes [92]. Furthermore, as electrode modifiers, CNTs may be readily functionalized with organic compounds, metal oxides, polymers, or metal nanoparticles, providing sensing materials for the electroanalysis of antidepressant drugs (Table 4).

**Figure 7:**
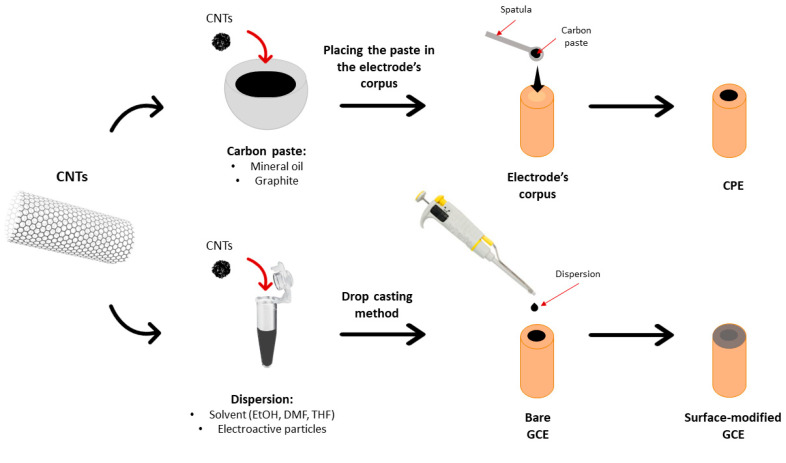
Electrode modification approaches realized with the use of CNTs.

Different electrode modification techniques were developed in order to obtain CNT-based sensors designed for antidepressant analysis. Typically, CNTs are utilized in the bulk modification of carbon paste electrodes (CPEs) and surface modification of solid-state electrodes (mainly GCEs). In the first approach, carbon paste has been used as a matrix for incorporating carbon nanotubes and other modifiers to improve the electrocatalytic properties of voltammetric sensors [93]. The surface modification has been mainly realized by drop casting a solution of CNTs to form a carbon nanotube film at the electrode surface (Figure 7). Recent studies have shown that CNT-modified electrodes can enhance the sensitivity of antidepressant determination, thereby improving the limit of detection in voltammetric measurements.

**Table 4:** CPE/CNTs-based electrochemical sensors of selected antidepressant drugs.

Electrode	Analyte	Technique	Linear Range	LOD	Sample	Ref.
^1^ CNTPE	Amitriptyline	DPV	0.0–30.0 µmol L^−1^	1.61 µmol L^−1^	Pharmaceutical formulation	[94]
^2^ MWCNTs/C/T/MCPE	Agomelatine	DPV	3.0 × 10^−9^–0.5 × 10^−6^ mol L^−1^ 1.0 × 10^−6^–5.0 × 10^−4^ mol L^−1^	5.26 × 10^−10^ mol L^−1^	Tablets, urine	[95]
^3^ ZnO-MWCNT/CPE	Citalopram	ASWV	0.012–1.54 µmol L^−1^	0.005 µmol L^−1^	Human serum, urine, pharmaceutical	[96]
^4^ MWCNTs/ZnO-NPs/CPE	Clonazepam	DPV	0.39–7.70 µg mL^−1^	0.17 µg mL^−1^	Drug products, human urine	[97]
	Desvenlafaxine		0.66–8.42 µg mL^−1^	0.28 µg mL^−1^	Drug products, human urine	
^5^ βCD-CNT-PE	Nifedipine	DPAdSV	4.77 × 10^−8^–2.00 × 10^−5^ mol L^−1^	1.48 × 10^−8^ mol L^−1^	Pharmaceutical formulation, biological fluid	[98]
^6^ CNT/CsMCPE/SDS	Sertaline	SWV	60 nM–15.0 µM	9.2 × 10^−9^ M	Biological fluid	[99]

^1^ Unmodified carbon nanotube paste electrode. ^2^ Multi-walled carbon nanotubes/Cellulose/Tween-modified CPE. ^3^ ZnO nanoparticle- and multi-walled carbon nanotube-modified CPE. ^4^ Carbon paste electrode modified with zinc oxide nanoparticles immobilized on multi-walled carbon nanotubes. ^5^ β-Cyclodextrin-modified multi-walled carbon nanotube paste electrode. ^6^ Cs multi-walled carbon nanotubes/sodium dodecyl sulfate-modified CPE. ASWV: Adsorptive Square-wave Voltammetry; DPAdSV: Differential Pulse Adsorptive Stripping Voltammetry.

#### 4.2.1. Carbon Paste Electrodes Based on CNTs

As shown in Table 4, carbon nanotube paste electrodes are widely applied for the electrochemical sensing of antidepressant drugs. For example, the unmodified carbon nanotube paste electrode based on MWCNTs and mineral oil was successfully applied in the DPV determination of amitriptyline in pharmaceutical samples in the presence of sulfuric acid as an electrolyte that provides a wide linear range (0.0 to 30.0 µmol L^−1^) and a low LOD equal to 1.61 µmol L^−1^ [94]. Concurrently, to improve electrochemical performance, the CNT paste electrodes are chemically modified by the implementation of various kinds of materials, such as chelating agents, ion exchangers, and metal nanoparticles. The multi-walled carbon nanotubes/Cellulose/Tween 40-modified carbon paste electrode was used as an innovative sensor for the sensitive determination of agomelatine in bulk solutions, urine samples, and pharmaceutical formulations. As a result of the strong synergistic effect of the applied modifiers, the proposed sensor exhibited two linear relationships between the anodic peak current and the agomelatine concentration from 1.0 × 10^−9^ to 0.5 × 10^−6^ mol L^−1^ and 1.0 × 10^−6^–5.0 × 10^−4^ mol L^−1^, with a detection limit of 5.26 × 10^−6^ mol L^−1^ [95]. With the introduction of ZnO nanoparticles and MWCNTs to the carbon paste electrode, the voltametric sensors characterized by favorable analytical performance for the determination of citalopram, desvenlafaxine, and clonazepam were achieved. In the case of the electro-determination of citalopram in human serum, urine, and pharmaceutical samples [96] using the adsorptive square-wave voltammetry (AdSWV) technique, the limit of detection and the linear range were found to be 0.005 and 0.012 and 1.54 μmol L^−1^, respectively. The CPE modified with zinc oxide nanoparticles immobilized on multi-walled carbon nanotubes (MWCNTs/ZnONPs/CPE) was exploited for the simultaneous determination of desvenlafaxine (DVS) and clonazepam (CLZ) [97] in its combined dosage form and in spiked human urine. The observed improvement in the analytical performance of MWCNTs/ZnONPs/CPE is attributed to the nanometric dimensions of ZnO-NPs and MWCNTs, which increase the effective surface area of the electrode. As a consequence, the linear range of 0.66–8.42 μg mL^−1^ and 0.39–7.70 μg mL^−1^ with an LOD of 0.280 μg mL^−1^ and 0.173 μg mL^−1^ for DVS and CLZ, using the DPV method, was achieved, respectively. In turn, the β-Cyclodextrin-modified multi-walled carbon nanotube paste electrode (βCD-CNT-PE) [98] and the Cs multi-walled carbon nanotubes/sodium dodecyl sulfate-modified carbon paste electrode (CNT/CsMCPE/SDS) [99] were developed for the sensitive and selective determination of nifedipine and sertraline, respectively. The superior performance of the β-CD-CNT-PE in the DPAdSV analysis of nifedipine is demonstrated by a linear range and a detection limit of 4.77 × 10^−8^ to 2.00 × 10^−5^ and 1.48 × 10^−8^ M, respectively. Due to some advantages such as good conductance of electricity and characteristic catalytic capacity, the CNT/CsMCPE/SDS was successfully applied in the electrochemical simultaneous estimation of sertraline and paracetamol in biological fluid with an LOD value equal to 9.2 × 10^−9^ M. 

#### 4.2.2. Glassy Carbon Electrodes Modified with CNTs

Glassy carbon electrodes modified with the film of CNTs constitute the most common working electrodes used for the electrochemical sensing of antidepressant drugs. The simplicity of surface modification of GCEs with drop casting methods creates the possibility to exploit the combination of different nanostructures with CNTs to improve the electrochemical performance toward target analytes (Table 5). 

For example, phosphorus-doped multi-walled carbon nanotubes (P-MWCNTs) were developed as a sensing element for amitriptyline (AMT) in two linear ranges, 0.50–10 µg mL^−1^ and 10–40 µg mL^−1^, with an LOD value equal to 0.15 µg mL^−1^ [100]. Selective determination of trace amounts of citalopram was obtained by the deposition of MWCNTs coated with a poly p-aminobenzene sulfonic acid/β-cyclodextrin (p-ABSA/β-CD) film [101] or a core–shell structured functionalized Fe_3_O_4_@SiO_2_ with Schiff base ligand/multi-walled carbon nanotubes suspension [102] on the surface of the GCE. The modified electrodes provided a considerable increase in sensitivity and improved the detection limit; thus, the nanomolar concentrations of citalopram in pharmaceuticals and human body fluids can be determined. Functionalized nanocluster-modified glassy carbon electrode (f-MWCNTs/GCE) showed a considerable improvement in the voltammetric response of clomipramine for which the linearity was observed in the concentration range of 1.45 × 10^−5^–4.52 × 10^−3^ mol L^−1^, and the LOD was equal to 1.315 × 10^−8^ g mL^−1^ [103]. By combining the attractive mechanical and electrical characteristics of CNTs with the unique properties of ionic liquid (IL) and nickel oxide nanoparticles (NiONPs), the MWCNTs/IL/NiONPs catalyzed the oxidation of clozapine, leading to an increase in the sensitivity of the sensor. As a result, DPAdS voltammograms were linear over the concentration range of 0.5–67 μM with a limit of detection of 0.052 µM [104]. An electrochemical sensor based on mercury nanoparticles (HgNPs) and a multi-walled carbon nanotube-modified glassy carbon electrode (HgNPs/MWCNTs/GCE) was developed for the determination of fluvoxamine in tablets and human urine [105]. Due to the co-effect of MWCNTs and HgNPs, the proposed electrode provided a wide dynamic range (0.020–1.750 µmol L^−1^) and a low limit of detection (0.01 µmol L^−1^). A nanocomposite consisting of amine-functionalized TiO_2_/MWCNTs and sodium dodecylsulfate for GCE modification was successfully applied in the SVW determination of olanzapine in tablets and blood serum samples [106]. The fabricated sensor exhibited high sensitivity and selectivity within the concentration ranges of 0.05–0.1 μM and 0.1–10 μM, achieving a detection limit of 8 nM. The glassy carbon electrode modified with a Nafion–MWCNT composite enables the highly sensitive detection of paroxetine through DPV, with a linear range of 0.1 to 2.5 µM and an LOD of 2.6 µg L^−1^ [107]. MWCNTs functionalized with different materials have also been described in the literature as efficient electrocatalysts for serotonin detection. With the application of a biocomposite based on the enzyme monoamine oxidase-A (MAO-A) immobilized by covalent binding on MWCNTs [108], serotonin sensors with an LOD value equal to 2 × 10^−7^ M were obtained. On the other hand, the electrochemical sensor based on the deposition of thin layer of mixture of multi-walled carbon nanotube–ionic liquid crystal and crown ether (CNTs-ILC)/crown [109], on the surface of the GCE, can be characterized by a wide linear dynamic range of 0.005 mmol L^−1^ to 100 mmol L^−1^ and an LOD of 2.02 × 10^−10^ mol L^−1^. The enhancement in the sensitivity of the simultaneous determination of serotonin and sertraline resulting from electrocatalytic performance and the elevated electroactive surface area of the composite of Fe_3_O_4_@MCM-48-SO_3_H nanoparticles [110] was also reported. Finally, GCEs modified with a composite consisting of electrodeposited chitosan and carboxylated MWCNTs (e-CS/MWCNTs/GCE) [111], as well as diazonium salt and single-walled carbon nanotubes composites (DS-SWCNT) [112], were applied as working electrodes for the determination of serotonin with an LOD value equal to 10 µmol L^−1^ and 4.6 nM, respectively. The preparation of a modified glassy carbon electrode based on Fe_3_O_4_@MCM-48-SO_3_H/MWCNTs [110] and the modification of MWCNT/GCE surface with gold nanoparticles and a nano-structured Ni(II)-levodopa film (Ni(II)-LD/AuNPs/MWCNT/GCE) [113] led to the development of a sensitive strategy for sertraline determination in human serum and urine. The uniform dispersion of cobalt nanoparticles (CoNPs) immobilized on the MWCNT surface ensured an efficient catalytic role for the electrochemical oxidation of thioridazine [114]. The fabricated CoNP/MWCNT/GCE created the possibility to determine sub-micromolar amounts of thioridazine in a dynamic range of 5.0 × 10^−7^ to 1.0 × 10^−4^ M with a detection limit of 5.0 × 10^−8^ M. The electro-oxidation and determination of trazodone can be obtained using MWCNTs/GCE [115] and titanium dioxide–carboxylated MWCNT-modified GCE (TiO_2_-cMWCNTs/GCE) [116]. The increase in the surface area of MWCNTs-TiO_2_ hybrid film enhanced both the adsorption of trazodone onto the electrode surface and the electron transfer during its electro-oxidation, leading to improved sensitivity and a lower LOD value (5.6 nM). A Nafion–carbon nanotube-modified glassy carbon electrode (NAF-CNT-GCE) [117], multi-wall carbon nanotube–ionic liquid gel-modified glassy carbon electrode (MWCNTs-RTIL/GCE) [118], and multi-wall carbon nanotube-modified carbon ionic liquid electrodes (MWCNT-CILE) [119] were successfully applied in the electrocatalytic oxidation and determination of venlafaxine in pharmaceutical formulation, urine, and blood serum. The benefits of the utilization of MWCNTs in the surface modification of GCEs can be expressed by the sub-micromolar detection limits, which were equal to 1.24 × 10^−8^ M, 1.69 × 10^−6^ M, and 0.47 µM for NAF-CNT-GCE, MWCNTs-RTIL/GCE, and MWCNT-CILE, respectively. 

#### 4.2.3. Other Sensors Based on CNTs

CNT surface-modified Au electrodes have been reported for antidepressant drug analysis as well (Table 6). Duloxetine-imprinted nanoparticles (DUL-nanoMIP) with SWCNTs embedded in the polytyramine film were deposited on the surface of the Au electrode, creating a chemosensor suitable for DUL clinical analysis. The linear dynamic range for the aforementioned sensor was between 10 pM and 676 nM, and the LOD was equal to 1.6 pM [120]. An amperometric sensor based on the immobilization of a bisphosphoramidate derivative (BMBPBP) and quantum dots on MWCNTs and their deposition on the surface of the Au electrode was successfully applied to the analysis of olanzapine in pharmaceutical and human serum samples [121]. The application of CuO nanoparticles deposited on MWCNTs in the surface modification of commercially available screen-printed electrodes (SPEs) for the detection of doxepin has been reported [122]. Due to the exceptional robust electrocatalytic activity of the modifiers to facilitate the doxepin oxidation reaction, the Cu/MWCNTs/SPE exhibited a wide linear range of 0.001–400.0 μM and a low LOD of 0.17 nM. 

## 5. Conclusions

Pharmacotherapy of mental disorders has never been more complex and essential. The increasing pace of life and omnipresent stress results in more and more of us being forced to reach for psychiatric drugs to return to normal functioning in everyday life. Due to the effectiveness of therapy and the potential side effects of the pharmaceuticals taken, it is extremely important to determine the appropriate therapeutic dose and carefully follow the doctor’s recommendations. The issue of quality control in the pharmaceutical industry and the possibility of monitoring the concentrations of active substances in patients’ body fluids to prevent overdose are extremely important. This review summarizes the current state of knowledge in the field of voltammetric determination of active substances used in popular psychiatric drugs, with particular emphasis on the use of carbon materials and their derivatives as working electrodes modifiers. The analysis of numerous articles published between 2002 and 2024 showed that scientific research on carbon material applications for highly sensitive psychiatric drug compound analysis is still an attractive research direction. Electrochemical sensors have gained a distinctive role in this field due to their simplicity, portability, and ease of use, with the development of new assays continuing to increase. These methods have also been demonstrated to be effective for quality control in the pharmaceutical industry and, in some instances, for monitoring drug concentrations in human body fluids.

Despite the accuracy, precision, and cost-effectiveness of voltammetric techniques, they are still not widely used on a large scale due to automation challenges. The complexity of electrode preparation, the need for precise experimental control, and the sensitivity of measurements to environmental factors make large-scale implementation difficult. As a result, voltammetry remains primarily used in small-scale, specialized applications such as laboratory research, the quality control of selected pharmaceutical products, or drug monitoring in specific clinical cases. Other difficulties are connected with the sample preparation process to remove undesirable interferents. Sample preparation procedures are still tedious and manually intensive protocols, especially considering environmental or biological samples in which organic compounds, such as antidepressant residues, are measured. Sample treatment is considered the most time-consuming and error-prone part of the analytical scheme. Overcoming these automation challenges could significantly increase the role of voltammetric sensors in routine pharmaceutical analysis and therapeutic drug monitoring.

## Figures and Tables

**Table 1:** GCE-based electrochemical sensors of selected antidepressant drugs.

Electrode	Analyte	Technique	Linear Range	LOD	Sample	Ref.
GCE	Opipramol	DPV	2 × 10^−6^–2 × 10^−4^	2.70 × 10^−7^	Serum, urine	[23]
		OSW		3.10 × 10^−7^		
GCE	Aripiprazole	LSV	0.1–5 mg L^−1^	50 µg L^−1^	Tablets, urine	[24]
		AdSV	4–40 µg L^−1^	1 µg L^−1^		
GCE	Aripiprazole	DPV	11.4–157 µM	6.16 µM	Tablets	[25]
		SWV		5.49 µM		
		DPAAdSV	0.221–13.6 µM	0.14 µM	Serum, urine	
		SWAAdSV		0.11 µM		
GCE	Citalopram	DPV	0.05–10.0 µM	0.036 µM	Pharmaceuticals, tap, river, and wastewater	[26]
			10.0–115.0 µM			
GCE	Quetiapine	DPV	4 × 10^−6^–2 × 10^−4^ mol L^−1^	4.01 × 10^−8^ mol L^−1^	Serum, urine	[27]
		OSWV		1.33 × 10^−7^ mol L^−1^		

DPV—Differential Pulse Voltammetry, OSWV—Osteryoung Square-wave Voltammetry, SWV—Square-wave Voltammetry, DPAAdSV—Differential Pulse Anodic Adsorptive Stripping Voltammetry, SWAAdSV—Square-wave Anodic Adsorptive Stripping Voltammetry.

**Table 2:** BDDE electrochemical sensors of selected antidepressant drugs.

Electrode	Analyte	Technique	Linear Range	LOD	Sample	Ref.
^1^ CP-BDDE	Duloxetine	DPV	0.030–0.333 µmol L^−1^	5.87 nmol L^−1^	Lake, river, and tap water	[31]
		SWV	0.10–12.4 µmol L^−1^	42 nmol L^−1^		
BDDE	Fluoxetine	SWV	3.2–162 µmol L^−1^	0.3 µmol L^−1^	Weight loss products in capsules	[32]
BDDE	Paroxetine	SWAdSV	7.0 × 10^−7^–3.5 × 10^−6^ M	6.95 × 10^−9^ M	Pharmaceutics	[33]

^1^ CP-BDDE—cathodically pretreated boron-doped diamond electrode.

**Table 3:** Graphene-based electrochemical sensors of selected antidepressant drugs.

Electrode	Analyte	Technique	Linear Range	LOD	Sample	Ref.
^1^ RGO/Co_3_O_4_/GCE	Serotonin	DPV	1–51 µM	48.7 nM	Serum	[57]
^2^ in situ-NiCo_2_O_4_@rGO/PGE	Venlafaxine	SWV	0.5–50 × 10^−8^ mol L^−1^	3.4 × 10^−9^ mol L^−1^	Pharmaceutical formulations, human plasma	[58]
^3^ RGO/PdNPs/GC	Desipramine	DPV	0.3–2.5 µmol L^−1^	1.04 nmol L^−1^	Urine	[59]
^4^ LI-AuNP-rGO/GCE	Alprazolam	DPAdCSV	0.001–0.10 mg L^−1^ 0.10–8.0 mg L^−1^	0.3 µg L^−1^	Beverage samples	[60]
^5^ rGO/PWA/PGE	Paroxetine	DPV	8.0 × 10^−9^–1.0 × 10^−6^ M	9.0 × 10^−10^ M	Tablets, human serum, urine	[61]
^6^ Ni(OH)_2_ × Gr-IL/CPE	Sulpiride	SWV	1.0 × 10^−9^–2.0 × 10^−4^ mol L^−1^	2.50 × 10^−10^ mol L^−1^	Biological fluids, pharmaceutical dosage form	[62]
^7^ AuNPs@GRP/GCE	Vortioxetine	AdsDPVs	0.1–1.0 µM 1.0–6.0 µM	0.050 µM	Tablets	[63]
^8^ Nb_2_CT_x_/NRGO/GCE	Fluoxetine	SWV	1.0–10 µM 10–100 µM	0.34 µM	Human serum and urine	[64]
^9^ GO/β-CD/PGE	Sulpiride	AdSSWV	1.0 × 10^−7^–5.0 × 10^−5^ M	2.83 × 10^−9^ M	Tablet, capsule, urine	[65]
^10^ GnPs-Naf/GCE	Quetiapine	DPAdSV	1 × 10^−7–^1 × 10^−5^ M	2.2 × 10^−8^ M	Tablets, human urine	[66]
^11^ MIP−Graphene Pt	Sertraline	DPV	1.0 × 10^−8^–1.0 × 10^−6^ mol L^−1^	7.0 × 10^−9^ mol L^−1^	Human serum	[67]
^12^ Au–PdNPs-GR/AuE	Citalopram	SWV	0.5–50 µM	0.049 µM	Tablet, plasma	[68]
^13^ GO/g-C_3_N_4_/GCE	Carbamazepine	amperometry	0.092–266 µM	10.5 nM	Human urine, pharmaceutical samples	[69]
^14^ rGO-D2	Amitriptyline	CV	1–80 µg/mL	1 ng/mL	-	[70]
^15^ SPCE/AGO-Cu	Flunitrazepam	DPV	0.4–140 µM	0.13 µM	Fruit juice	[71]
^16^ HSA-FeM-rGO/SPCE	Imipramine	DPV	10–756 ng/mL	4 ± 2 ng/mL	Plasma, serum	[72]
^17^ Cu-MOF/SNDGr/PGE	Sertraline	DPV	0.05–2.67 µM	0.038 µM	Tablet, human serum	[73]
^18^ AgVO_3_/c-GO/GCE	Sertraline	DPV	0–1600 µM	25 μM	Pharmaceutical sample	[74]
^19^ MIP/Gr-SPCE	Sertraline	SWV	5.0 × 10^−9^–7.5 × 10^−7^ M	1.99 × 10^−9^ M	Tablet, human serum	[75]
^20^ C/T/Pd/MCPE	Vilazodone	DPV	2.5 × 10^−8^–2 × 10^−4^ M	8 × 10^−10^ M	Pharmaceutical dosage forms, urine	[76]
^21^ nano-MIP/G-CP	Fluoxetine	DPV	0.006–0.1 µM	0.0015 µM	Plasma, pharmaceutical samples	[77]
^22^ CPE	Duloxetine	SW-AdASV	1.0 × 10^−8^–1.0 × 10^−6^ M	3.0 × 10^−9^ M	Pharmaceutical formulation, human serum	[78]
^23^ NiCACP	Escitalopram	DPV	1.0 × 10^−6^–7.0 × 10^−5^ M	2.0 × 10^−7^ M	Dosage form, urine	[79]
^24^ MIP-CPE	Quetiapine	SWV	1.6 × 10^−8^–2.5 × 10^−6^ M	5.04 × 10^−9^ M	Pharmaceutical formulation, human urine	[80]
^25^ Al_2_O_3_NP-CPE	Aripiprazole	SWAdSV	0.03–8.0 µM	0.006 µM	Pharmaceutical formulations, human serum	[81]

^1^ Reduced graphene oxide/cobalt oxide nanocomposite on a glassy carbon electrode. ^2^ Electrodeposited NiCo_2_O_4_ microspheres anchored on a reduced graphene oxide platform. ^3^ Glassy carbon electrode modified by reduced graphene oxide modified with palladium nanoparticles. ^4^ Laser-induced gold nanoparticles and reduced graphene oxide-modified glassy carbon electrode. ^5^ Reduced graphene oxide/phosphotungstic acid on pencil graphite electrode. ^6^ Carbon paste electrode consisting of β-Ni(OH)_2_, reduced graphene oxide, and ionic liquid. ^7^ Gold nanoparticles/graphene nanocomposite on glassy carbon electrode. ^8^ Niobium carbide (Nb_2_CT_x_) and nitrogen-doped reduced graphene oxide (NRGO)-hybridized electrodes. ^9^ Pyrolytic graphite electrode modified with graphene oxide (GO) and β-cyclodextrin (β-CD). ^10^ Graphene nanoplatelet-modified GCE. ^11^ Platinum electrode modified with graphene nanoparticles and molecular imprinting polymer. ^12^ Gold–palladium bimetallic nanoparticles decorated with graphene-modified gold electrode. ^13^ GO/g-C_3_N_4_–graphene oxide and graphitic carbon nitride composite film-modified electrode. ^14^ Reduced graphene oxide–7′-methoxy-[1,1′-binaphthalen]-7-ol. ^15^ Screen-printed carbon electrode modified with amine-functionalized graphene oxide sheets reinforced through Cu nanoparticles. ^16^ Human serum albumin/iron molybdate/reduced graphene oxide. ^17^ Copper-based metal–organic framework on/in the heteroatom-doped graphene/pencil graphite electrode. ^18^ Carboxylated graphene oxide-modified glassy carbon electrodes and silver vanadate nanoparticles. ^19^ Molecularly imprinted polymer graphene-modified screen-printed electrode. ^20^ Cellulose/nanotitanium oxide/nanopalladium chloride-modified carbon paste electrode. ^21^ Graphene/molecularly imprinted polymer-modified carbon paste electrode. ^22^ Carbon paste electrode, ^23^ nickel nanoparticle-modified chloranil carbon paste sensor. ^24^ Molecularly imprinted polymer-modified carbon paste electrode. ^25^ Carbon paste electrode modified with aluminum oxide nanoparticles.

**Table 5:** GCE/CNTs-based electrochemical sensors of selected antidepressant drugs.

Electrode	Analyte	Technique	Linear Range	LOD	Sample	Ref.
^1^ P-MWCNTs/GCE	Amitriptyline	AdSV	0.50–10 µg mL^−1^ 10–40 µg mL^−1^	0.15 µg mL^−1^	Pharmaceutical tablets	[100]
^2^ p(*p-ABSA*)/β-CD/MWCNT/GC	Citalopram	DPV	90 nM–1 µM 1–11 µM 11–100 µM	44 nM	Pharmaceutical, human body fluids	[101]
^3^ Fe_3_O_4_@[(EtO)3Si-L]/MWCNTs/GCE	Citalopram	DPV	0.02–62 µM	32.2 nM	Human blood serum, pharmaceuticals	[102]
^4^ f-MWCNTs/GCE	Clomipramine	DP AAdSV	1.45 × 10^−5^–4.52 × 10^−3^ mol L^−1^	1.315 × 10^−8^ g mL^−1^	Drug tablets	[103]
^5^ MWCNT-IL/NiONPs/GCE	Clozapine	DP AdSV	0.5–67 µM	0.052 µM	Human serum, pharmaceuticals	[104]
	Sertraline		0.21–85 µM	0.047 µM		
^6^ HgNPs/MWCNTs/GCE	Fluvoxamine	DPV	0.020–1.750 µmol L^−1^	0.01 µmol L^−1^	Tablets, urine	[105]
^7^ ISS-NH_2_-TiO_2_-MWCNTs/GCE	Olanzapine	SWV	0.05–0.1 µM 0.1–10 µM	8 nM	Tablets, blood serum	[106]
^8^ Nafion/MWCNTs/GCE	Paroxetine	DPV	0.1–2.5 µM	8 nM	Tablets, urine	[107]
^9^ MWCNTs/MAO-A/GCE	Serotonin	DPV	5.67 × 10^−7^–2.26 × 10^−6^ M	2 × 10^−7^ M	-	[108]
^10^ GC/CNTs-ILC/Crown	Serotonin	DPV	0.005–100 µM	2.02 × 10^−10^ mol L^−1^	Human serum,	[109]
^11^ Fe_3_O_4_@MCM-48-SO_3_H/MWCNTs/GCE	Serotonin	DPV	0.05–100 µM	0.015 µM	Human serum, urine	[110]
	Sertraline		0.1–85 µM	0.025 µM		
^12^ e-CS/MWCNTs/GCE	Serotonin	DPV	9–1000 µmol L^−1^	10 µmol L^−1^	Human saliva	[111]
^13^ DS-SWCNTs/CFMEA	Serotonin	DPV	0.07–0.9 µm	4.6 nM	Mouse striatum	[112]
^14^ Ni(II)-LD/AuNPs/MWCNT/GCE	Sertraline	DPV	0.05–5.5 µM	95 nM	Human serum	[113]
^15^ CoNP/MWCNT/GCE	Thioridazine	DPV	5.0 × 10^−7^–1.0 × 10^−4^ M	5.0 × 10^−8^ M	Human blood serum,	[114]
^16^ MWCNTs/GCE	Trazodone	DPV	0.2–10 µM	24 nM	Urine	[115]
^17^ TiO_2_-cMWCNTs/GCE	Trazodone	DPASV	6–100 nM 100–1000 nM	5 nM	Pharmaceutical formulation, human serum	[116]
^18^ NAF-CNT-GCE	Venlafaxine	DPAdSV	3.81 × 10^−8^–6.22 × 10^−5^ M	1.24 × 10^−8^ M	Pharmaceutical formulation, urine, blood serum	[117]
^19^ MWCNTs-RTIL/GC	Venlafaxine	SWV	2.0 × 10^−6^–2.0 × 10^−3^ M	1.69 × 10^−6^	Pharmaceutical	[118]
^20^ MWCNT-CILE	Venlafaxine	CV	10.0–500.0 µM	0.47 µM	Pharmaceutical formulation, urine, blood serum	[119]

^1^ Phosphorus-doped multi-walled carbon nanotube-modified GCE. ^2^ Multi-walled carbon nanotube/poly(*p*-aminobenzene sulfonic acid)/β-cyclodextrin-modified GCE. ^3^ Core–shell structured functionalized Fe_3_O_4_@SiO_2_ with Shiff base ligand/multi-walled carbon nanotube-modified GCE. ^4^ Functionalized MWCNT nano-cluster-modified GCE. ^5^ Multi-walled carbon nanotube/ionic liquid/nickel oxide nanoparticle-modified GCE. ^6^ Mercury nanoparticles multi-walled carbon nanotube-modified GCE. ^7^ In situ surfactant-modified amine-functionalized TiO_2_/multi-walled carbon nanotube-modified GCE. ^8^ Multi-walled carbon nanotubes with Nafion-modified GCE. ^9^ Monoamine oxidase-A/multi-walled carbon nanotube-modified GCE. ^10^ Multi-walled carbon nanotube–ionic liquid crystal and crown ether-modified GCE. ^11^ Fe_3_O_4_@MCM-48-SO_3_H/multi-wall carbon nanotube-modified GCE. ^12^ Electrodeposited chitosan/carboxylated multi-walled carbon nanotube-modified GCE. ^13^ Diazonium single-walled carbon nanotube composite-modified carbon fiber microelectrode array. ^14^ Nano-structured Ni-levodopa/gold nanoparticles enriched multi-walled carbon nanotube-modified GCE. ^15^ Co-nanoparticles/multi-walled carbon nanotube-modified GCE. ^16^ Multi-walled carbon nanotube-modified GCE. ^17^ Titanium dioxide–carboxylated multi-walled carbon nanotube-modified GCE. ^18^ Nafion–carbon nanotube composite GCE. ^19^ Multi-walled carbon nanotubes and a room-temperature ionic liquid-modified GCE. ^20^ Multi-walled carbon nanotube-modified carbon ionic liquid electrode.

**Table 6:** Other CNT-based electrochemical sensors of selected antidepressant drugs.

Electrode	Analyte	Technique	Linear Range	LOD	Sample	Ref.
^1^ nanoMIPs-SWCNTs@polytyramine film-coated Au electrode	Duloxetine	DPV	10 pM–676 nM	1.6 pM	Human plasma	[120]
^2^ BMBPBP/CdS-QDs/MWCNTs	Olanzapine	Amerometric	20 nM–100 µM	6nM	Pharmaceutical, human serum	[121]
^3^ CuO/MWCNTs/SPE	Doxepin	Amperometric	0.001–400 µM	0.17 nM	Tablets, urine samples	[122]

^1^ Nano-molecularly imprinted polymer single-walled carbon nanotube @polytyramine film-coated Au-disk electrode. ^2^ 1,4-bis(N-methyl)-benzene-bis(N-phenyl,N-benzoylphosphoramidate)/CdS-quantum dots/multi-walled carbon nanotube-modified gold electrode. ^3^ Copper oxide nanoparticles and multi-walled carbon nanotube-modified screen-printed electrode.

## Data Availability

No new data were created or analyzed in this study.
